# Entropion congénital bilatéral: à propos d'un cas

**DOI:** 10.11604/pamj.2014.18.20.4172

**Published:** 2014-05-06

**Authors:** Hakima Elouarradi, Rajae Daoudi

**Affiliations:** 1 Université Mohammed V Souissi, Service d'Ophtalmologie A de l'hôpital des spécialités, Centre hospitalier universitaire, Rabat, Maroc

**Keywords:** Entropion congénital, ulcération cornéenne, Congenital entropion, corneal ulceration

## Image en medicine

Enfant âgée de 2 ans et demi, emmené par ses parents pour prise en charge d'un entropion congénital bilatéral isolé (A). L'examen ophtalmologique n'a pas noté d'ulcération cornéenne au niveau des deux yeux. Notre technique chirurgicale était simple par trois points de sutures en U au niveau du pli palpébral inférieur (B et C), réalisés sous anesthésie générale, avec un bon résultat fonctionnel et esthétique (D). L'entropion congénital est en rapport avec une hypertrophie du muscle orbiculaire prétarsal et pré septal. Il s'agit de la rotation de la marge palpébrale vers le globe; les cils frottant alors sur la cornée provoquent une kératite, plus ou moins gênante et sévère qui domine la prise de décision thérapeutique. L'entropion congénital régresse le plus souvent spontanément lors de la croissance. Il existe deux formes physiopathologiques principales. Le diagnostic différentiel peut se faire avec l’épiblépharon. Le diagnostic positif est difficile chez un enfant éveillé; et peut être évoqué devant une irritation oculaire, photophobie, et surtout une ulcération cornéenne. Le traitement est chirurgical lorsque la kératite risque de provoquer des séquelles oculaires par la réalisation de sutures éversantes avec résection d'une languette myocutanée en regard. Le pronostic est bon surtout si le diagnostic et la prise an charge sont précoces.

**Figure 1:**
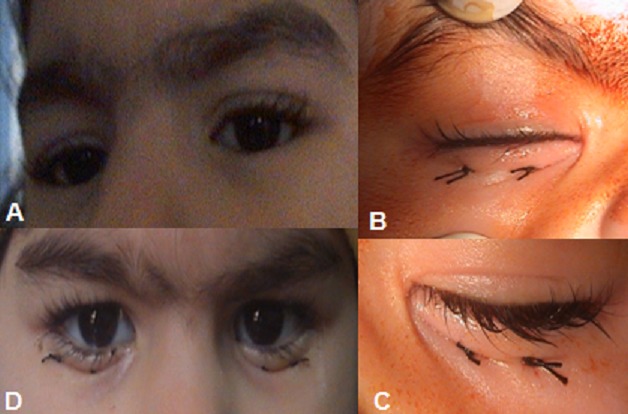
(A) Entropion congénital bilatéral; (B et C) Aspect per opératoire: technique chirurgicale avec 3 points de sutures éversantes au niveau de la paupière inférieure; (D) Bon résultat postopératoire

